# Radical Formation in Sugar-Derived Acetals under Solvent-Free Conditions

**DOI:** 10.3390/molecules26195897

**Published:** 2021-09-29

**Authors:** Aleksandra A. Wróblewska, H. Y. Vincent Ching, Jurrie Noordijk, Stefaan M. A. De Wildeman, Katrien V. Bernaerts

**Affiliations:** 1 Faculty of Science and Engineering, Biobased Materials, Maastricht University, P.O. Box 616, 6200 MD Maastricht, The Netherlands; olaelwroblo@gmail.com (A.A.W.); jurrie.noordijk@maastrichtuniversity.nl (J.N.); sdw@b4plastics.com (S.M.A.D.W.); 2 Department of Chemistry, University of Antwerp, Universiteitsplein 1, B-2610 Wilrijk, Belgium; HongYueVincent.Ching@uantwerpen.be

**Keywords:** polycondensation, polymerization, EPR spectroscopy, GalX, polyamide, radical decomposition, sugar derived monomers

## Abstract

The degradation of acetal derivatives of the diethylester of galactarate (GalX) was investigated by electron paramagnetic resonance (EPR) spectroscopy in the context of solvent-free, high-temperature reactions like polycondensations. It was demonstrated that less substituted cyclic acetals are prone to undergo radical degradation at higher temperatures as a result of hydrogen abstraction. The EPR observations were supported by the synthesis of GalX based polyamides via ester-amide exchange-type polycondensations in solvent-free conditions at high temperatures in the presence and in the absence of radical inhibitors. The radical degradation can be offset by the addition of a radical inhibitor. The radical is probably formed on the methylene unit between the oxygen atoms and subsequently undergoes a rearrangement.

## 1. Introduction

Acetal moieties constitute a recurring motif in chemical synthesis whether in the context of organic synthesis as labile protective groups [1]. or, more recently, in polymer synthesis [2,3]. A wide variety of acetal protective groups have been used to protect carbohydrate-based polyols prior to their polymerization, resulting in functional bio-based polymers with tunable properties [4]. For example, the incorporation of biacetilized mannarates, glucarates or galactarates into polymers elevates their glass transition region, suppresses crystallization of polymeric domains and lowers melting temperatures [5,6]. These properties can be translated to material properties and result in transparent polymers, which sustain their shapes at higher temperatures and simultaneously are easier to process due to their lower melting points. Furthermore, the protective groups can be selectively removed [7] leading to OH^−^ functionalized polymers, which are widely used in coatings, dynamic networks or high added-value materials for biomedical applications, etc. 

**Figure 1:**
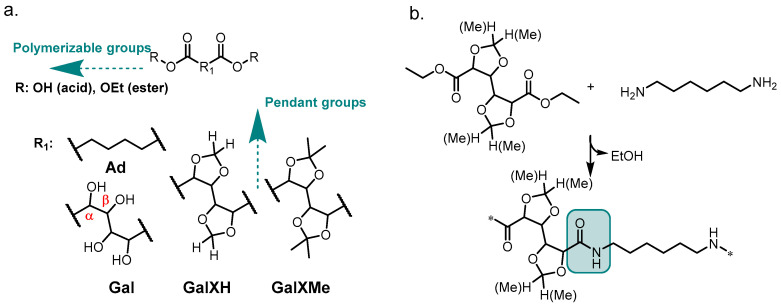
(**a**) The general structure of the investigated acetals (GalXH and GalXMe) and adipate motif (Ad); (**b**) the polymer obtained by reacting diethyl esters of GalXH and GalXMe with 1,6–hexamethylenediamine.

The incorporation of acetal motifs into polyesters is well-documented; however, until recently, attempts to incorporate them into polyamides have been rather scarce. Polyamides (PAs) are a wide-spread type of polymers with renowned performance and chemical resistance. Typical polycondensation reactions to obtain PAs utilize the diacid (Figure 1 with R=H) form of the molecule. 

Upon reaction with diamines, polyamides are formed via intermediate salt formation. During the reaction, high temperatures (above 200 °C) are applied, releasing water and driving the equilibrium to the right. All these conditions (acid, water and heat) are incompatible with acetal chemistry. More precisely, substituted cyclic acetals (Figure 1a) can easily undergo acidic hydrolysis because stabilized tertiary carbocations are formed as intermediates [8]. This aspect has always been troubling for polyamide synthesis from GalX and consequently the majority of reported syntheses have been conducted in solution [9,10,11] through active ester methods utilizing, e.g., toxic pentachlorophenyl esters [3,12] and significant amounts of solvents, which are incompatible with the sustainable character of the monomers and could never find wide-spread application. In contrast, solvent-free polymer synthesis methods have received growing attention because of increasing environmental awareness and a greater focus on green chemistry principles [13], as well as economic aspects, e.g., the elimination of solvents and product isolation steps. Recent developments showed that in-melt polycondensation of cyclic acetal-bearing diacid monomers is possible if certain conditions are met [6]. Less substituted dioxolane-bearing molecules such as GalXH have been favored, due to their higher stabilities in acidic conditions. On the other hand, more substituted acetal rings (e.g., *i*-propylidene acetals like GalXMe) have been considered less stable and therefore neglected in polymer solvent-free methods using diacid monomers. However, we recently observed reversed stabilities under non-acidic conditions [5,13].

A recently published series of articles exhaustively describes the synthesis of polyamides from the diethyl esters (instead of the diacids) of sugar-derived cyclic acetal monomers (Figure 1a R=Et) in the melt [5,14]. Polymers with broader dispersities (branching) or even crosslinked networks were obtained with the less-substituted acetal monomer (GalXH) while better defined polymers were obtained with the more-substituted monomer (GalXMe). A similar polymerization method, but in solution, has been reported by Ogata et al. for diethyl adipate (Figure 1a Ad) and non-protected diethyl galactarate (Figure 1a Gal) [9,10,11,15]. They observed that polyhydroxy diethyl esters are activated towards diamines by the presence of oxygen atoms in the *α* and/or *β* position (see Figure 1a). The published findings were never translated to solvent-free methods and were never widely explored for the whole class of similar sugar-derived molecules. In addition, a similar dispersity trend has also been reported for polyester synthesis in the melt, which is less thermally demanding, using GalXH and GalXMe monomers, where broadening of the dispersity with GalXH was also observed and was attributed to transesterification [16]. This similarity suggests that the observed trend could be valid for all solvent-free polycondensations involving GalXH.

The aim of the present study is to carefully investigate the stability of acetal-containing monomers, mainly focusing on reaction conditions during melt polycondensation. The thermal stability of the GalX monomers has been elucidated using electron paramagnetic resonance (EPR) and supported by polymerization experiments in the melt (Figure 1b), which has shed new light on the underlying mechanism governing the observed behavior, thus aiding the development of solvent-free experiments, and later, material design.

## 2. Results and Discussion

### 2.1. Radical Degradation Study of the Acetal Fragments

**Figure 2:**
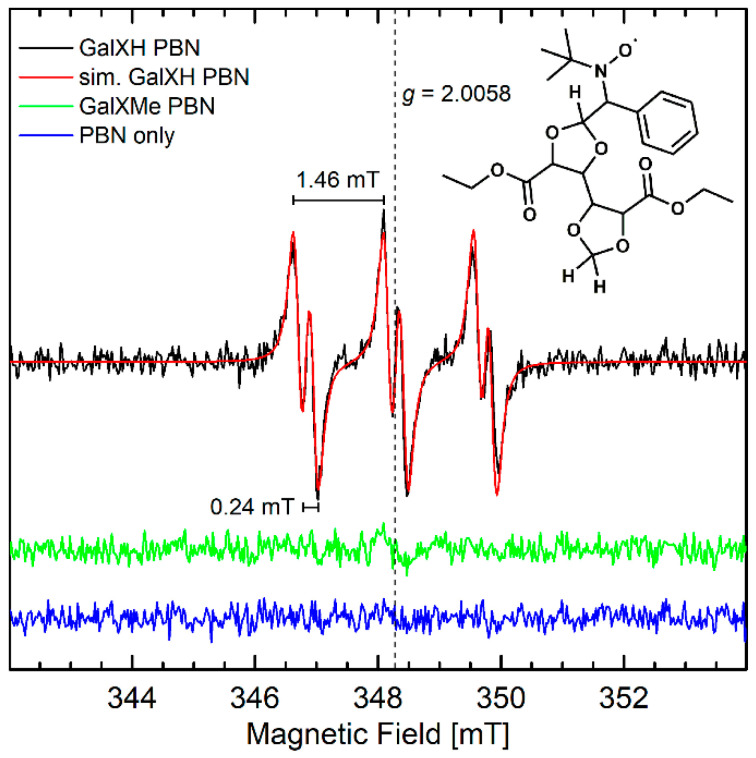
cw X-band (~9.7 GHz) solution EPR spectra of GalXH and GalXMe samples that had been heated in the presence of PBN and subsequently dissolved in toluene measured at room temperature using 10 mW microwave power, 0.1 mT modulation amplitude and 100 kHz modulation frequency. Simulation of the GalXH spectrum is shown in red, as well as the chemical structure of a possible spin-trapped adduct.

Since the lower stability of less-substituted acetal rings in neutral reaction conditions cannot be explained by an ionic mechanism via intermediate carbocation formation [8], it was hypothesized that it proceeds via a radical pathway. To verify the proposed radical branching/cross-linking mechanism, EPR investigations were conducted. No paramagnetic species were detected when neat GalXH or GalXMe samples were heated and EPR spectra were subsequently recorded at room temperature or at 100 K. This meant either radical intermediates were not present, or they were highly reactive and could not be observed within the timeframe of the EPR measurements. Consequently, *N*-tert-butyl-α-phenyl nitrone (PBN) was added to the heating experiments as a spin trap. Spin traps react with radical intermediates, forming longer-living radical adducts that can be detected by EPR [17]. The EPR spectra of the adducts are characterized by the isotropic g-value (*g*_iso_), isotropic hyperfine couplings of the nitroxide nitrogen (*A*_N iso_), the *α*-proton (*A*_H*α* iso_), and in some cases also other protons, which are dependent on the spin-trapped radical. The mixtures were only heated to 140 °C because the concerted thermal decomposition of PBN at this temperature is still relatively slow [18]. A clear room temperature EPR signal was observed for the heated GalXH/PBN mixture that was subsequently dissolved in toluene, while, under the same conditions, no signals were observed for the heated GalXMe/PBN mixture or PBN alone (Figure 2). The observed signal is a triplet of doublets with *g*_iso_ = 2.0058, *A*_N iso_ = 1.46 mT, and *A*_H*α* iso_ = 0.24 mT which are consistent with a carbon-centered radical trapped by PBN [17].

Since the only difference between GalXH and GalXMe are the methyl groups at the 2-position of the 1,3-dioxlane moiety, it is likely that the unpaired electron is centered at this position in the GalXH radical. We hypothesize that a radical is generated by hydrogen atom abstraction. The corresponding PBN-trapped dioxolanyl radical adduct is depicted in Figure 2. Furthermore, it should be noted that cyclic acetal radicals can undergo rearrangements, such as β-scission [19] giving aldehyde moieties. In our previous research on GalXH [8] aldehyde moieties were observed, which further supports the assignment of the dioxolanyl radical.

From the radical degradation studies, it appears that the *i*-propylidene acetals are resistant to thermal degradation via the radical mechanism, since they do not possess hydrogen atoms in the 2-position of the 1,3-dioxlane moiety for abstraction. Consequently, GalXMe can be freely used in the solvent-free high-temperature polymerization conditions, at least if acids are not present in a significant amount since they degrade *i*-propylidene acetals via a cationic mechanism [8]. The utilization of methylene acetals should be avoided. It is worth mentioning that multiple studies in the past used this type of acetal for polymerization, but the reports only include solution polymerization [3,20]. This choice could be motivated by the observed degradation and could be avoided if another acetal was chosen.

### 2.2. Polymerization

The relevance of the findings to the field were verified by conducting the melt polymerization of the diethyl esters of the two presented acetals (GalXH and GalXMe) with 1,6-hexamethylene diamine (HMDA), resulting in polyamides (Figure 1b). Such reactions require temperatures above 200 °C, mechanical stirring, and the removal of by-products (alcohol). During the reactions two parameters were investigated: the concentration of a radical inhibitor (Irganox 1330—a sterically hindered phenolic antioxidant) and the structure of the acetal. If radical degradation mechanisms were interfering with the polymerization, narrower dispersities of the final polymers would be expected in the presence of the inhibitor than in the absence of the inhibitor. Therefore, the polymerization reactions were evaluated based on the dispersity of the product. The theoretical value of the dispersity for polycondensates is 2 [21], however, in practice the value might vary due to limitations of the theory [22]. as well as side reactions [8]. Typically, if cyclization of polymeric chains is observed, or in the case of limited conversions (< 100%), the dispersity drops below 2. High dispersity typically means that side reactions occur that lead to increased functionality and consequently to branching or cross-linking at high conversions.

The reactions were performed using GalXH (**PA1** and **PA2** in Table 1) or GalXMe (**PA3** and **PA4** in Table 1), and diethyl esters with (**PA2** and **PA4** in Table 1) or without (**PA1** and **PA3** in Table 1) the radical inhibitor. The molecular weight distribution of GalXH polymers strongly depended on the presence of the inhibitor and, even in that case, an increase with respect to the generally accepted value was observed. The molecular weight of GalXMe polymers was not affected by the addition of the inhibitor, where a dispersity value close to 2 was observed in both entries.

**Figure 3:**
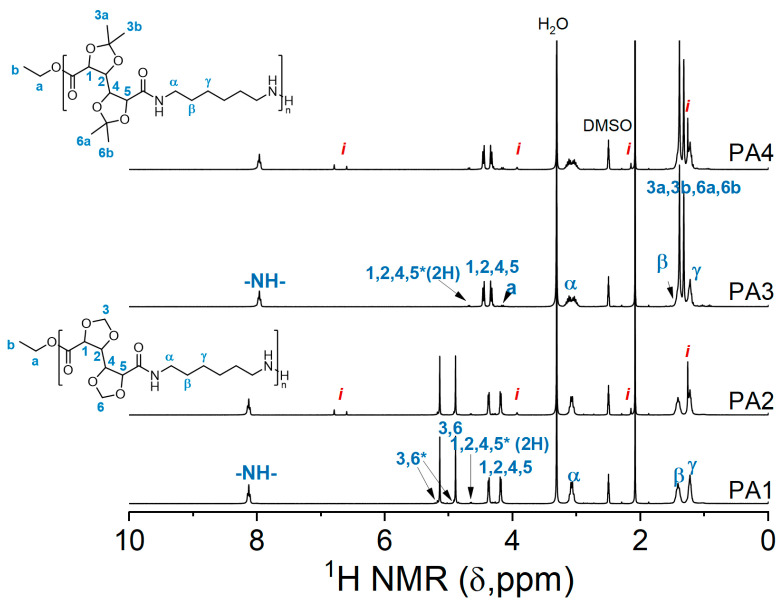
^1^H NMR spectra of **PA1**, **PA2**, **PA3** and **PA4**. The resonances of end groups are marked with a star “*” and resonances of Irganox 1330 with a letter “*i*”.

NMR analysis revealed that, structurally, the polymers with and without the inhibitor are similar (Figure 3.). Furthermore, numerical analysis of the products according to the Carothers theory provided data about the molecular weight, degree of polymerization, and the extent of polymerization. The molecular weight of the polymers, though lower than obtained by GPC, follows the same trend. **PA1** has a higher degree of polymerization than the other polymers. The extent of polymerization is 95.0–96.3% with the highest value achieved for **PA1**. This experiment showed that in the case of GalXMe the addition of an inhibitor is not necessary because it only introduces impurities without any significant improvement in dispersity. For GalXH, on the other hand, the addition of the inhibitor is necessary since it improves the polymer dispersity by the reduction in radical side reactions.

**Figure 4:**
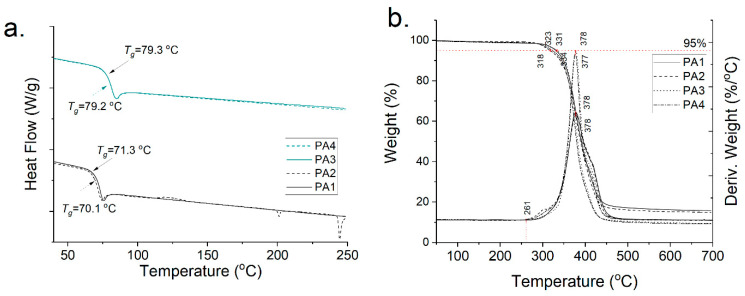
Thermal profiles of **PA1**, **PA2**, **PA3** and **PA4** (**a**) DSC curves and (**b**) TGA profiles.

The polyamides **PA1**–**4** were further analyzed via DSC and TGA. The DSC curves confirm that in the case of GalXMe (**PA3**,**4** in Figure 4a) the addition of the inhibitor does not cause any distinguishable changes in the polymer. The *T*_g_’s of **PA3** and **PA4** are 79.3 °C and 79.2 °C, respectively, and the curves do not show any additional thermal events which might point towards side reactions with an inhibitor. In contrast the GalXH polyamides (**PA1**,**2** in Figure 4a) show the opposite. The thermal profile of the polyamide obtained with the addition of the inhibitor (**PA2**) shows multiple thermal events which are hypothesized to originate from the reactions between the inhibitor and acetal fragmentation. Upon the addition of the inhibitor the *T*_g_ drops slightly from 71.3 °C to 70.1 °C which is in line with the fact that **PA1** has higher molar mass in comparison to **PA2**.

The TGA analysis (Figure 4b) does not show any influence of the inhibitor on the degradation profiles of the polymers. They all start to degrade above 260 °C in an inert atmosphere and show 5% weight loss above 300 °C. All curves show that even with a rigorous drying step there is still insignificant weight loss observed at 100 °C, which is in line with our previous findings regarding water sorption of GalX polyamides [14].

## 3. Experimental Section

### 3.1. Materials and Methods

#### 3.1.1. Materials

Diethyl 2,3:4,5-di-*O*-isopropylidene-galactarate > 99% (GalXMe) and diethyl 2,3:4,5-di-*O*-methylene-galactarate > 99% (GalXH) were supplied by Royal Cosun (Roosendaal, the Netherlands). 1,6-hexamethylene diamine (HMDA) 98%, Irganox 1330 and NaTFA were purchased from Sigma-Aldrich (Zwijndrecht, the Netherlands)and used as supplied. 1,1,1,3,3,3-hexafluoro-2-propanol (HFIP) and DMSO-*d_6_* were purchased from Acros Organics (The Hague, the Netherlands)and used as supplied. N-tert-butyl-α-phenyl nitrone (PBN) was purchased from TCI Europe N. V. (Zwijndrecht, Belgium).

#### 3.1.2. Methods

Molecular weight of the polyamides was determined via gel permeation chromatography (GPC) supplied by Polymer Standards Service GmbH (PSS, Mainz, Germany). The polymers were dissolved in 1,1,1,3,3,3-hexafluoroisopropanol (HFIP) with 0.019% NaTFA salt. The sample for GPC measurement was prepared by dissolving 5.0 mg of the polymer in 1.5 mL of the solvent. The solutions were filtered over a 0.2 μm PTFE syringe filter before injection. The GPC apparatus was calibrated with poly(methyl methacrylate) standards. Two PFG combination medium microcolumns with 7 µm particle size (4.6 × 250 mm, separation range 100–1.000.000 Da) and a precolumn PFG combination medium with 7 µm particle size (4.6 × 30 mm) with refractive index detector (RI) were used in order to determine molecular weight and dispersities.

^1^H NMR spectra were recorded in DMSO-*d_6_* on a Bruker Avance III HD Nanobay 300 MHz NMR spectrometer (Bruker Biospin GmbH, Rheinstetten, Germany). 

Differential scanning calorimetry (DSC). DSC was performed on a TA Instruments Discovery DSC 250 (TA Instruments, New Castle, United States) equipped with a refrigerated cooling system (RCS). The samples were measured in Tzero pans with perforated Tzero hermetic lids to allow a nitrogen atmosphere around the sample. DSC thermograms were recorded with a heating rate of 10 °C min^−1^. Only experimental data obtained from the second heating step are reported.

Thermal stabilities of the prepared polyamides were determined using thermogravimetric analysis (TGA) (TA Instruments Q500, TA Instruments, New Castle, DE, United States). Approximately 10 mg of the material was heated at 10 °C/min from 25 °C to 700 °C in a nitrogen atmosphere.

Electron paramagnetic resonance (EPR). 

EPR sample preparation.

Neat GalXH or GalXMe (~50 mg) were loaded inside 4 mm quartz EPR tubes. The samples were heated at 200 °C under a nitrogen atmosphere. After 15 min the samples were rapidly cooled in liquid nitrogen and were transferred directly to the spectrometer. Mixtures of the spin-trap *N*-tert-Butyl-α-phenyl nitrone (30 mg, 0.17 mmol) with and without GalXH (45 mg, 0.15 mmol) or GalXMe (52 mg, 0.15 mmol) were heated at 140 °C under a nitrogen atmosphere. After 15 min the mixtures were cooled to room temperature and then each was dissolved in 300 µL of toluene. Aliquots of each solution were transferred to 2 mm capillaries for EPR measurements.

EPR

Room temperature EPR measurements were carried out using a Bruker E580 Elexys spectrometer (Bruker Biospin GmbH, Rheinstetten, Germany). The EPR spectra were recorded at X-band microwave frequency (~9.7 GHz) in continuous-wave (CW) mode with microwave power of 10 mW, 0.1 mT modulation amplitude and 100 kHz modulation frequency. Cryogenic temperature EPR measurements were carried out using a Bruker ESP300E spectrometer (Bruker Biospin GmbH, Rheinstetten, Germany) at X-band microwave frequency (~9.45 GHz). The EPR spectra were simulated with Matlab2018b (Mathworks, Natick, MA, United States) using the EasySpin-6.0 module, [23].

#### 3.1.3. Synthesis of Polymers

A typical procedure adapted from Wroblewska et al. [5] is given below.

Synthesis of poly(hexamethylene-2,3:4,5-di-*O*-iso-propylidene-galactaramid) with 5 wt% inhibitor.

To a 100 mL three-necked round bottom flask (equipped with a vacuum-tight mechanical stirrer, a Vigreux column and a distillation condenser) diethyl 2,3:4,5-di-*O*-isopropylidene-galactarate (6.9276 g, 20 mmol), 1,6-hexamethylene diamine (2.3716 g, 20.4 mmol, slight excess taking into account the 98% purity of the starting product), and Irganox 1330 as an inhibitor (0.4626 g, 5 wt%) were added and slowly heated under nitrogen to 200–220 °C. After all ethanol had been fully distilled off (+/− 1.5 h), vacuum was applied for 3 h. The crude product was obtained as a yellowish material.

^1^H NMR (300 MHz, DMSO-*d_6_*) δ (ppm): 6.68 (2H, -NH-, s), 4.55 (2H, -CH-O-, m), 4.36 (2H, -CH-O-, m), 4.28 (4H, -CH_2_-NH-, t), 1.58 (4H, -C*H_2_*-CH_2_, HMDA, m), 1.40 (6H, CH_3_, GalX, s), 1.30 (6H, -CH_3_, GalX, s). 1.26 (4H, -C*H_2_*-CH_2_-, HMDA, m).

## 4. Conclusions

Sugar-based cyclic acetal monomers are interesting monomers for solvent-free melt polycondensation reactions. They are abundant and easily obtained from waste streams, however the structure of the monomer should be carefully matched to the polymerization conditions. It was confirmed by EPR measurements that methylene acetals have the tendency to form radicals via thermally induced abstraction of hydrogen atoms and therefore participate in undesired side reactions. The degradation can be potentially offset using radical inhibitors, which allows control over polymerization of methylene acetals, but is redundant during the polymerization of *i*-propylidene acetals since they are not subject to radical degradation. Although these findings are based only on polyamide synthesis, which requires more demanding temperatures, they are applicable to other (melt) polycondensation reactions in general.

## Figures and Tables

**Table 1:** The list of prepared polymers and their properties.

			Polymer					
Diethyl Acetal Type	Diamine Type	Inhibitor ^b^ (wt%)	Polymer Code	GPC ^c^		NMR ^d^		
				*M_n_* (kg·mol^−1^)	*Ð*	*M_n,NMR_* (g·mol^−1^)	*DP (−)*	*p_GalX_ (%)*
GalXH	HMDA ^a^	0	**PA1**	23.7	4.53	8300	26	96.3
GalXH	HMDA ^a^	5	**PA2**	16.3	2.93	6900	22	95.6
GalXMe	HMDA ^a^	0	**PA3**	15.0	1.83	8200	22	95.7
GalXMe	HMDA ^a^	5	**PA4**	14.0	1.88	7000	19	95.0

^a^ 1,6-hexamethylenediamine, ^b^ wt% relative to both monomers, ^c^ GPC with RI detection in 1,1,1,3,3,3-hexafluoro-2-propanol/0.019%. NaTFA calibrated with poly (methyl methacrylate) standards. ^d^ The molecular mass *M_n,NMR_*, degree of polymerization (DP) and extent of the polymerization (p) were calculated based on the NMR resonances of reacted GalX 1,2,4,5 at 3.27–4.39 ppm and end group resonances of 1,2,3,4 (marked with * in Figure 3) at 4.66–4.65 ppm.

## Data Availability

The data presented in this study are available on request from the corresponding author.

## Sample Availability

Samples of the compounds **PA1**–**PA4** are available from the authors.
